# The function of the thyroid gland in patients with multi-drug resistant tuberculosis

**DOI:** 10.1186/s13756-017-0238-4

**Published:** 2017-08-16

**Authors:** S. L. Matveyeva, O. S. Shevchenko, O. O. Pogorelova

**Affiliations:** Department of phtisiology and pulmonology, Kharkiv national medical university, Nauky lane, 4, Kharkiv, 61162 Ukraine

**Keywords:** Tuberculosis, Thyroid, Antituberculosis chemotherapy, Hypothyroidism

## Abstract

**Background:**

Multidrug-resistant tuberculosis (MDRTB) remains a health problem for many countries in the world. The share of MDRTB is 10–30% among newly diagnosed cases and 20–70% among relapses and treatment failure. The aim of the study is to define the side effects of second line drugs used in the treatment of MDRTB on thyroid function.

**Methods:**

In 30 patients with multidrug resistant tuberculosis, echostructure of thyroid was studied by ultrasound imaging method. Indices of thyroid function: plasma levels of free thyroxin, thyroid stimulating hormone were studied before chemotherapy initiated, at the end of intensive phase and after the treatment finished.

**Results:**

Decreasing of thyroid function under antituberculosis chemotherapy was approved. Monitoring and correction of thyroid function during antituberculosis chemotherapy was suggested.

**Conclusion:**

Patients with MDRTB taking ethionamide and PAS are at increased risk for hypothyroidism and goiter, and therefore require monitoring of thyroid function at all stages of antituberculosis chemotherapy for its timely correction.

## Background

Multidrug-resistant tuberculosis (MDR TB) remains a health problem for many countries in the world [1, 2]. The share of MDR TB is 10–30% among newly diagnosed cases and 20–70% among relapses and treatment failure [3]. Treatment of these cases demands a 20-month chemotherapy regimen which includes 8 months of intensive phase. Drugs used to treat MDR TB can cause a number of adverse reactions. Undesirable effects on the thyroid gland are not seen but the side effects of para-aminosalicylic acid and ethionamide and their combinations are well known. The side effects of these drugs are well documented, however, are often overlooked [4–6]. Real study is undertaken as a prospective one to illuminate unclear points of the problem previously discussed.

The aim of the study is to define the side effects of second line drugs used in the treatment of MDR TB on thyroid function.

## Methods

Clinical observations were carried out at the Kharkiv Regional TB Dispensary. Thirty patients with multidrug-resistant tuberculosis were examined. The diagnosis was established in the presence of resistance of mycobacterium tuberculosis, isolated from the sputum samples of patients, to isoniazid and rifampicin. The average age of the patients was 38.57 ± 10.9 years, male subjects prevailed (3: 1). The patients received individual chemotherapy with the inclusion of ethionamide and PAS.

The study was conducted in accordance with international standard ethical standards and issued using appropriate protocols and obtaining informed consent from all patients included in the study.

The functional state of the thyroid gland was assessed by the results of the study of the thyroid echostructure and the determination of serum levels of free thyroxine (T4) and the thyroid-stimulating hormone of the pituitary (TSH) before the start of therapy, as well as at the end of the intensive phase and at the end of treatment. These indicators were selected in accordance with modern generally accepted screening standards for the detection of thyroid function. Subclinical dysfunction of TSH level was diagnosed at the significance of more than 4.5 μIU / ml even at normal level of T4. Hypothyroidism is separated into either overt or subclinical disease. That diagnosis is determined on the basis of the TSH laboratory blood tests. The normal range of TSH concentration falls between 0.45–4.5 mU/L.

Patients with mildly underactive (subclinical) thyroid have TSH levels of 4.5-10 mU/L. Patients with levels greater than 10 mU/L are considered to have overt hypothyroidism and should be treated with medication.

Thyroid gland was visualized using the diagnostic ultrasound device SSF-240a, production of Toshiba Medical Systems.

Statistical processing of the obtained data was carried out by the method of variation statistics with the help of the standardized package of calculations of Microsoft Excel XP. The probability of divergence of the mean values ​​was determined by Student test. The critical level of significance (P) in testing statistical hypotheses was assumed to be 0.05.

## Results

A pathology of the thyroid gland was revealed in the study of its ultrasound; 2 (6,67%) - its goiter transformation (in the both cases mixed form), in 5 (16,67%) – diffusely enlarged thyroid with the presence of hypoechoic micronodules (1–6 mm) with surrounding echogenic septations and in others -signs of diffused pathology in the form of granularity (mosaic) of its structure, containing areas of normal, increased and decreased echogenicity, which is characteristic of autoimmune thyroiditis. The study of free thyroxine level revealed its low-normal concentration in patients with tuberculosis at the beginning of treatment (12,71 ± 0,98 pmol/l) with a reliable decreasing of this hormone by the end of the intensive phase (10,43 ± 0,85 pmol/l) and an even lower decrease after completion of treatment (8,33 ± 0,87 pmol/l) (Table 1).

**Table 1:** The levels of thyroid hormones in patients with multidrug resistant tuberculosis (MDR TB)

Criterion	Referent significance	Before the start of the treatment	Two months after start of the treatment	At the end of the treatment
Free T_4_ (pmol/l)	9.0–22.0	12,71 ± 0,98	10,43 ± 0,85 P_1,2 ≥_ 0.5	8,33 ± 0,87 P_1,3 ≤_ 0.5
TSH (μIU / ml)	0.4–4.0	1.29 ± 0.08	1.80 ± 0.04 P_1,2 ≥_ 0.5	4.8 ± 0.14 P_1,3 ≤_ 0.5

The mean level of TSH increased at the end of the intensive care phase (from 1.29 ± 0.08 to 1.80 ± 0.04 μIU / ml) with an even greater increase towards the end of treatment (up to 4.8 ± 0.14 μIU / ml). The data obtained indicates a significant weakening of thyroid function in MDR TB patients under the influence of individual chemotherapy with the inclusion of ethionamide and PAS. The obtained data allows making a conclusion about the suppressive effect of anti-tuberculosis drugs on the hormonal function of the thyroid gland of multidrug-resistant tuberculosis patients. Of the 30 patients (18 men and 12 women), 10% developed clinical hypothyroidism, which was diagnosed by increasing the level of TSH and lowering the level of free T4. Two patients (6.6%) developed goiter, one of which was euthyroid and one hypothyroid with the decreasing the level of free T4 (6.11 pmol/l.) and increasing the level of TSH (17,0 μIU / ml). Hypothyroidism was well compensated for with 50 μg of L-thyroxine with normalization of hormone level: decreasing of TSH up to 3.67 μIU / ml and increasing of free T4 up to11.43 pmol/l. The duration of goiter development was in average 8 months (range 6–13 months). The most noticeable symptoms of hypothyroidism were anemia, decreased tendon reflexes, hoarseness, puffiness, weakness and constipation. A patient with clinical hypothyroidism received simultaneously ethionamide and PAS.

## Discussion

The effect of anti-tuberculosis chemotherapy on thyroid function, especially such as ethionamide and PAS, has been shown in a number of studies and was explained by a decrease in the systemic blood flow of thyroxine-binding protein, resulting in a decrease in the thyroid hormone content in the blood. When analyzing the thyroid function in multidrug-resistant patients with a treatment period of 8 months, 11% of patients developed hypothyroidism with chemotherapy, and 7,4% with goiter. To compensate for hypothyroidism, patients with tuberculosis were prescribed L-thyroxine for 3 months or more. After the end of the course of chemotherapy, they should give up thyroxine. The appearance of goiter and hypothyroidism were detected under the influence of PAS at 8 months from the beginning of antituberculosis therapy. The administration of thyroxin at a dose of 100 mcg for 4 weeks resulted in a decrease in goiter and restoration of thyroid function.

Subclinical, or mild, hypothyroidism (mildly underactive thyroid), also called early-stage hypothyroidism, is a condition in which thyrotropin (TSH) levels have started to increase in response to an early decline in T4 levels in the thyroid. However, blood tests for T4 are still normal. The patient may have mild symptoms (usually slight fatigue) or none at all. Each year, about 2–5% of people with subclinical thyroid go on to develop overt hypothyroidism. In time new case-finding of subclinical hypothyroidism and its correction prevents developing of overt hypothyroidism and its correction strengthens immunity and improves treatment response toward antituberculosis chemotherapy.

## Conclusions

Thus, patients with MRD TB taking ethionamide and PAS are at increased risk for hypothyroidism and goiter, and therefore require monitoring of thyroid function at all stages of antituberculosis chemotherapy for its timely correction.

## Recommendations

The patients with MRD TB taking ethionamide and PAS submit monitoring of thyroid function by ultrasound and checking the levels of free thyroxine and thyroid stimulating hormone before the start of therapy, as well as at the end of the intensive phase and at the end of treatment.

## Abbreviations

MDRTB: multi-drug resistant tuberculosis
PAS: Para-aminosalicylic acidA
TSH: thyroid stimulating hormone
